# Maternal and Neonatal Outcomes Associated With Cesarean Versus Vaginal Delivery in the United States: A Population-Based Retrospective Cross-Sectional Analysis of CDC Natality Public Use Data (2017-2020)

**DOI:** 10.7759/cureus.105143

**Published:** 2026-03-12

**Authors:** Emasenyie Isikwei, Joseph E Igetei, Oluwatobi O Asade, Delete Ebere-Bank, Anita S Asamoah-Twum, Akinyele Oladimeji, Iyiola A Oyelese, Okelue E Okobi

**Affiliations:** 1 Family Medicine, Larkin Community Hospital Palm Springs Campus, Hialeah, USA; 2 General Medicine, International University of the Health Sciences, Basseterre, KNA; 3 Family Medicine, Southern Regional Area Health Education Center (AHEC), Fayetteville, USA; 4 Pediatrics, John H. Stroger, Jr. Hospital of Cook County, Chicago, USA; 5 General Practice, NHS Education for Scotland (NES), Glasgow, GBR; 6 Family Medicine, Obafemi Awolowo University, Ile-Ife, NGA; 7 General Practice, Lagos University Teaching Hospital, Lagos, NGA; 8 Family Medicine, IMG Research Academy and Consulting LLC, Homestead, USA

**Keywords:** cesarean delivery, maternal morbidity, natality data, neonatal outcomes, united states, vaginal delivery

## Abstract

Background: Cesarean delivery is a common obstetric procedure in the United States, yet concerns remain regarding its association with maternal and neonatal outcomes at the population level. Understanding outcome patterns by mode of delivery is important for informing clinical practice and public health planning.

Objective: To examine population-level associations between mode of delivery and severe maternal morbidity (blood transfusion, uterine rupture, unplanned hysterectomy, or maternal intensive care unit admission) and adverse neonatal outcomes (neonatal intensive care unit admission, assisted ventilation, or infant transfer) among live births in the United States.

Methods: This retrospective cross-sectional study analyzed the Centers for Disease Control and Prevention (CDC) Natality public use microdata from 2017 to 2020, including all recorded live births during the study period. The mode of delivery was categorized as cesarean or vaginal. Severe maternal morbidity and adverse neonatal outcomes were defined using composite indicators derived from reported clinical variables. Multivariable logistic regression was used to estimate adjusted odds ratios (aORs) while controlling for maternal demographic characteristics, clinical comorbidities, obstetric factors, and year of delivery. Robust standard errors were applied.

Results: Among the 15,035,328 births included in the analysis, severe maternal morbidity occurred in 32,630 (0.32%) vaginal deliveries compared with 55,463 (1.16%) cesarean deliveries (p < 0.001). The composite adverse neonatal outcome was observed in 770,413 (7.52%) vaginal deliveries and 903,895 (18.88%) cesarean deliveries (p < 0.001). In multivariable analyses adjusting for maternal demographic characteristics, clinical comorbidities, obstetric factors, and year of delivery, cesarean delivery was associated with higher odds of severe maternal morbidity compared with vaginal delivery (aOR, 2.99; 95% CI, 2.94-3.04). Cesarean delivery was also associated with higher odds of adverse neonatal outcomes (aOR, 2.19; 95% CI, 2.19-2.20). Preterm birth demonstrated a strong association with adverse neonatal outcomes (aOR, 13.87; 95% CI, 13.81-13.93).

Conclusion: Cesarean delivery was associated with higher maternal and neonatal morbidity in this national analysis. These associations likely reflect, in part, underlying obstetric complexity and high-risk clinical conditions that contribute both to the decision for cesarean delivery and to adverse outcomes. These findings emphasize the importance of careful clinical decision-making and continued efforts to improve maternal and neonatal care in high-risk pregnancies.

## Introduction

Childbirth is among the most common causes of hospitalization in the United States and a very important factor in developing and shaping maternal and neonatal health outcomes [1]. Cesarean delivery has significantly increased over the past decades; it is now among the most common surgical procedures that are carried out all over the world [2]. The changes in the obstetric practice, maternal characteristics, medico-legal aspects, and the change in clinical guidelines in the United States have led to cesarean section being almost one in every three live births [3,4]. Although cesarean section is a life-saving procedure when performed on the basis of medical necessity, its growing popularity has brought up the worries as to the possible short and long-term health effects on both the mothers and babies [5].

Vaginal birth is normally regarded as the physiologic birth approach, and there is a shorter maternal recovery period, a lesser possibility of surgical complications, and less healthcare expenses [6,7]. On the contrary, cesarean section, which is a significant abdominal operation, has its risks such as infection, bleeding, thromboembolism, and extended hospitalization [8]. In the case of neonates, cesarean delivery has been associated with respiratory problems, late start of breastfeeding, and dysregulated initial microbial colonization, which can affect immune response development [9,10]. Nonetheless, cesarean birth is able to drastically decrease maternal and neonatal morbidity and mortality in cases of obstructed labor, fetal distress, placenta previa, and other obstetric crises [11]. The role is dual in nature, which highlights the necessity to find the right balance between adequate clinical application and overuse [12].

Although numerous studies have compared cesarean and vaginal births, there are still gaps in the knowledge of the impact of mode of delivery on the population level of maternal and neonatal outcomes in a variety of demographic and clinical settings [13]. The association between the mode of delivery and health outcomes may vary depending on maternal factors (age, parity, comorbid conditions, and socioeconomic status), and neonatal factors (gestational age and birth weight) [14,15]. Furthermore, there have been numerous cases in the past where studies have been constrained by single-center designs, small sample sizes, or targeted subgroups; hence, they cannot serve as a generalization of the article on the general population of the United States [16]. Knowledge of the maternal and neonatal outcomes of cesarean section versus vaginal birth is necessary to inform clinical decision-making, optimize obstetric care, and influence the health policy of the population [17,18].

The study will employ nationally representative Centers for Disease Control and Prevention (CDC) Natality publicly available microdata from 2017 to 2020. The CDC Natality publicly available data offers detailed and standardized data on all recorded live births of the United States in terms of maternal demographics, prenatal care characteristics, method of delivery, and significant maternal and neonatal outcomes [19]. The use of years of Natality data allows for a strong evaluation of trends and associations and considers the changes in obstetric practices over the years [20]. The objective of the study is to examine population-level associations between mode of delivery and severe maternal morbidity (blood transfusion, uterine rupture, unplanned hysterectomy, or maternal intensive care unit admission) and adverse neonatal outcomes (neonatal intensive care unit (NICU) admission, assisted ventilation, or infant transfer) among live births in the United States using nationally representative CDC Natality publicly available microdata from 2017 to 2020. As the campaign against unsafe and evidence-based childbirth practices and unnecessary cesarean sections is intensified, a population-based analysis is important in determining outcome patterns and groups at risk.

## Materials and methods

Study design and data source

This study used a retrospective cross-sectional design based on data from the United States National Center for Health Statistics Natality publicly available microdata files for the years 2017 through 2020 [21]. The Natality files include population-level records of all registered live births occurring in the United States and contain standardized information on maternal demographics, pregnancy characteristics, delivery method, and neonatal outcomes derived from birth certificates. The use of multiple consecutive years allowed assessment of maternal and neonatal outcomes associated with cesarean delivery using consistent variable definitions across the study period.

Study population

The study population consisted of all live births recorded in the Natality Public Use files from 2017 to 2020. This period was selected to ensure consistent variable definitions and data structure across years, as several key maternal and neonatal variables underwent coding and reporting changes in earlier files, and to avoid heterogeneity introduced by subsequent revisions to data collection and reporting practices. Analyses were limited to hospital-based deliveries with a documented mode of delivery. Births with an unknown mode of delivery were excluded. No additional exclusions were applied based on maternal age, gestational age, or other clinical characteristics in order to maintain population-level coverage. After applying the inclusion criteria, the final analytic sample included 15,035,328 births.

Variables and measures

The primary exposure was mode of delivery, categorized as cesarean delivery or vaginal delivery using the National Center for Health Statistics delivery method recode. Maternal outcomes were assessed using a composite measure of severe maternal morbidity defined by the occurrence of any of the following events during the delivery hospitalization, maternal blood transfusion, uterine rupture, unplanned hysterectomy, or admission to a maternal intensive care unit. Neonatal outcomes were evaluated using a composite adverse neonatal outcome defined by NICU admission, assisted ventilation immediately after birth, assisted ventilation for more than six hours, or infant transfer to another facility following delivery. Composite outcome variables were constructed as binary variables representing the presence of at least one component event and were computed using Stata version 18 (StataCorp LLC, College Station, TX, USA) [22].

Additional neonatal indicators included the five-minute Apgar score and birth weight. A low five-minute Apgar score was defined as below 7, and low birth weight was defined as birth weight less than 2500 grams. These variables were examined descriptively to characterize neonatal status by mode of delivery but were not modeled as independent outcomes because they represent intermediate physiologic indicators rather than clinically severe outcomes requiring escalation of care, which were the focus of the composite neonatal endpoint. Gestational age was measured using the obstetric estimate in completed weeks, and preterm birth was defined using the National Center for Health Statistics obstetric estimate recode for births occurring before 37 weeks of gestation.

Covariates included maternal age in years, maternal education categorized into four levels with a separate category for unknown education, smoking during pregnancy, pre-pregnancy diabetes, gestational diabetes, chronic hypertension, gestational hypertension, eclampsia, prior cesarean delivery, and calendar year of delivery. Maternal race and Hispanic origin were analyzed as separate variables, consistent with the structure of the CDC Natality Public Use dataset. Race was categorized as White individuals, Black individuals, American Indian or Alaska Native individuals, Asian individuals, Native Hawaiian or Other Pacific Islander individuals, and individuals of more than one race. Hispanic origin was coded independently as Hispanic or non-Hispanic. Because the Natality Public Use files do not provide a combined race-ethnicity variable, these variables were analyzed separately rather than as mutually exclusive racial/ethnic groups.

Missing data

Missing data were minimal across study variables. Five-minute Apgar score was missing for approximately 0.4% of births, preterm status was missing 0.1%, and Hispanic origin was missing for 0.9% of records due to incomplete reporting. Missing values were retained as missing, and analyses were conducted using complete case analysis for each outcome without imputation.

Statistical analysis

Descriptive statistics were used to summarize maternal and neonatal characteristics. Group comparisons by the outcome variable were conducted using t-tests for continuous variables and chi-square tests for categorical variables. Multivariable logistic regression models were fitted to estimate associations between cesarean delivery and maternal and neonatal outcomes while adjusting for maternal demographic and clinical covariates. Calendar year was included as a categorical variable to account for temporal variation across the study period. Multicollinearity among covariates was assessed using variance inflation factors, which ranged from 1.00 to 3.08, with a mean value of 1.35, indicating no evidence of problematic collinearity. All analyses were performed using Stata version 18 [22].

Ethical considerations

The Natality Public Use microdata files are publicly available and fully de-identified. The use of these data does not constitute human subjects research under applicable federal regulations. Institutional review board approval and informed consent were not required.

## Results

Table 1 presents maternal demographic, obstetric, and neonatal characteristics by mode of delivery among live births in the United States from 2017 to 2020. Characteristics are shown separately for vaginal and cesarean deliveries to describe differences in maternal and neonatal profiles between delivery modes.

**Table 1:** Maternal and Neonatal Characteristics by Mode of Delivery Values are presented as mean ± standard deviation or n (%). Continuous variables were compared using t-tests, and categorical variables were compared using chi-square tests. Percentages represent row percentages. Sample sizes vary due to missing data. Five-minute Apgar score was available for 10,200,502 vaginal and 4,778,721 cesarean deliveries; preterm birth status for 10,241,394 vaginal and 4,786,160 cesarean deliveries; and Hispanic origin for 10,155,369 vaginal and 4,745,518 cesarean deliveries. Analyses were performed using Stata version 18 (StataCorp LLC, College Station, TX, USA) [22].

Characteristic	Vaginal delivery (N = 10,248,204)	Cesarean delivery (N = 4,787,124)	Chi-square/t-test	p-value
Maternal age, years (mean ± SD)	28.52 ± 5.74	30.16 ± 5.81	t = -510.0	<0.001
Gestational age, weeks (mean ± SD)	38.69 ± 2.43	37.94 ± 2.63	t = 539.5	<0.001
Birth weight, grams (mean ± SD)	3292.4 ± 556.5	3200.2 ± 718.6	t = 271.9	<0.001
Five-minute Apgar score (mean ± SD)	8.83 ± 0.74	8.68 ± 0.96	t = 329.0	<0.001
Maternal race, n (%)				
White mothers	7,602,398 (69.0)	3,414,864 (31.0)	χ^2^ = 23,000	<0.001
Black mothers	1,549,486 (64.2)	863,790 (35.8)		
American Indian or Alaska Native mothers	102,848 (71.2)	41,626 (28.8)		
Asian mothers	677,342 (67.2)	330,842 (32.8)		
Native Hawaiian or Other Pacific Islander mothers	34,472 (69.3)	15,243 (30.7)		
More than one race	281,658 (70.0)	120,759 (30.0)		
Hispanic origin, n (%)				
Non-Hispanic	7,715,418 (68.1)	3,621,600 (31.9)	χ^2^ = 208.4	<0.001
Hispanic	2,439,951 (68.5)	1,123,918 (31.5)		
Maternal education, n (%)				
Less than high school	1,332,303 (71.6)	527,855 (28.4)	χ^2^ = 16,000	<0.001
High school graduate	2,656,814 (68.9)	1,197,648 (31.1)		
Some college or associate degree	2,806,036 (67.0)	1,384,108 (33.0)		
Bachelor’s degree or higher	3,314,022 (67.3)	1,613,381 (32.7)		
Unknown	139,029 (68.4)	64,132 (31.6)		
Smoking during pregnancy, n (%)				
No	9,633,078 (68.3)	4,470,542 (31.7)	χ^2^ = 2,100	<0.001
Yes	615,126 (66.0)	316,582 (34.0)		
Pre-pregnancy diabetes, n (%)				
No	10,188,636 (68.4)	4,700,883 (31.6)	χ^2^ = 51,000	<0.001
Yes	59,568 (40.9)	86,241 (59.1)		
Gestational diabetes, n (%)				
No	9,644,932 (68.9)	4,351,563 (31.1)	χ^2^ = 52,000	<0.001
Yes	603,272 (58.1)	435,561 (41.9)		
Chronic hypertension, n (%)				
No	10,087,574 (68.6)	4,621,611 (31.4)	χ^2^ = 55,000	<0.001
Yes	160,630 (49.3)	165,513 (50.7)		
Gestational hypertension, n (%)				
No	9,613,634 (69.1)	4,304,671 (30.9)	χ^2^ = 72,000	<0.001
Yes	634,570 (56.8)	482,453 (43.2)		
Eclampsia, n (%)				
No	10,228,892 (68.2)	4,765,705 (31.8)	χ^2^ = 8,100	<0.001
Yes	19,312 (47.4)	21,419 (52.6)		
Prior cesarean delivery, n (%)				
No	9,934,565 (78.2)	2,763,683 (21.8)	χ^2^ = 3,800,000	<0.001
Yes	313,639 (13.4)	2,023,441 (86.6)		
Preterm birth, n (%)				
Term (≥37 weeks)	9,511,122 (70.4)	4,004,444 (29.6)	χ^2^ = 310,000	<0.001
Preterm (<37 weeks)	730,272 (48.3)	781,716 (51.7)		

The findings from Table 1 indicate statistically significant differences in maternal and neonatal characteristics by mode of delivery. Mean maternal age was higher among cesarean deliveries at 30.16 (5.81) years compared with 28.52 (5.74) years among vaginal deliveries. Mean gestational age was lower among cesarean deliveries at 37.94 (2.63) weeks compared with 38.69 (2.43) weeks among vaginal deliveries. Mean birth weight was lower among cesarean deliveries at 3200.2 (718.6) grams compared with 3292.4 (556.5) grams among vaginal deliveries. The mean five-minute Apgar score was lower among cesarean deliveries at 8.68 (0.96) compared with 8.83 (0.74) among vaginal deliveries.

Maternal race differed by delivery mode. Among White mothers, 7,602,398 (69.0%) had vaginal deliveries and 3,414,864 (31.0%) had cesarean deliveries. Among Black mothers, 1,549,486 (64.2%) delivered vaginally and 863,790 (35.8%) delivered by cesarean. Among American Indian or Alaska Native mothers, 102,848 (71.2%) had vaginal deliveries and 41,626 (28.8%) had cesarean deliveries. Among Asian mothers, 677,342 (67.2%) delivered vaginally and 330,842 (32.8%) delivered by cesarean. Among Native Hawaiian or Other Pacific Islander mothers, 34,472 (69.3%) had vaginal deliveries and 15,243 (30.7%) had cesarean deliveries. Among mothers reporting more than one race, 281,658 (70.0%) delivered vaginally and 120,759 (30.0%) delivered by cesarean.

Differences were observed by Hispanic origin. Among non-Hispanic mothers, 7,715,418 (68.1%) had vaginal deliveries and 3,621,600 (31.9%) had cesarean deliveries. Among Hispanic mothers, 2,439,951 (68.5%) delivered vaginally and 1,123,918 (31.5%) delivered by cesarean.

Maternal educational attainment varied across delivery modes. Mothers with less than a high school education had 1,332,303 (71.6%) vaginal deliveries and 527,855 (28.4%) cesarean deliveries. High school graduates had 2,656,814 (68.9%) vaginal deliveries and 1,197,648 (31.1%) cesarean deliveries. Mothers with some college education or an associate degree had 2,806,036 (67.0%) vaginal deliveries and 1,384,108 (33.0%) cesarean deliveries. Mothers with a bachelor’s degree or higher had 3,314,022 (67.3%) vaginal deliveries and 1,613,381 (32.7%) cesarean deliveries. Mothers with unknown education had 139,029 (68.4%) vaginal deliveries and 64,132 (31.6%) cesarean deliveries.

Smoking during pregnancy differed by delivery mode. Among mothers who did not smoke during pregnancy, 9,633,078 (68.3%) had vaginal deliveries and 4,470,542 (31.7%) had cesarean deliveries. Among mothers who reported smoking, 615,126 (66.0%) delivered vaginally and 316,582 (34.0%) delivered by cesarean.

Pre-pregnancy diabetes was more frequent among cesarean deliveries. Among mothers without pre-pregnancy diabetes, 10,188,636 (68.4%) had vaginal deliveries and 4,700,883 (31.6%) had cesarean deliveries. Among mothers with pre-pregnancy diabetes, 59,568 (40.9%) delivered vaginally and 86,241 (59.1%) delivered by cesarean. Gestational diabetes also differed by delivery mode, with 603,272 (58.1%) vaginal deliveries and 435,561 (41.9%) cesarean deliveries among affected mothers.

Hypertensive disorders differed across delivery modes. Among mothers without chronic hypertension, 10,087,574 (68.6%) had vaginal deliveries and 4,621,611 (31.4%) had cesarean deliveries. Among mothers with chronic hypertension, 160,630 (49.3%) delivered vaginally and 165,513 (50.7%) delivered by cesarean. Among mothers with gestational hypertension, 634,570 (56.8%) delivered vaginally and 482,453 (43.2%) delivered by cesarean. Among mothers with eclampsia, 19,312 (47.4%) had vaginal deliveries and 21,419 (52.6%) had cesarean deliveries.

Prior cesarean delivery differed substantially by delivery mode. Among mothers without a prior cesarean delivery, 9,934,565 (78.2%) had vaginal deliveries and 2,763,683 (21.8%) had cesarean deliveries. Among mothers with a prior cesarean delivery, 313,639 (13.4%) delivered vaginally and 2,023,441 (86.6%) delivered by cesarean.

Preterm birth differed by delivery mode. Among term births, 9,511,122 (70.4%) were vaginal deliveries and 4,004,444 (29.6%) were cesarean deliveries. Among preterm births, 730,272 (48.3%) were vaginal deliveries and 781,716 (51.7%) were cesarean deliveries.

**Figure 1:**
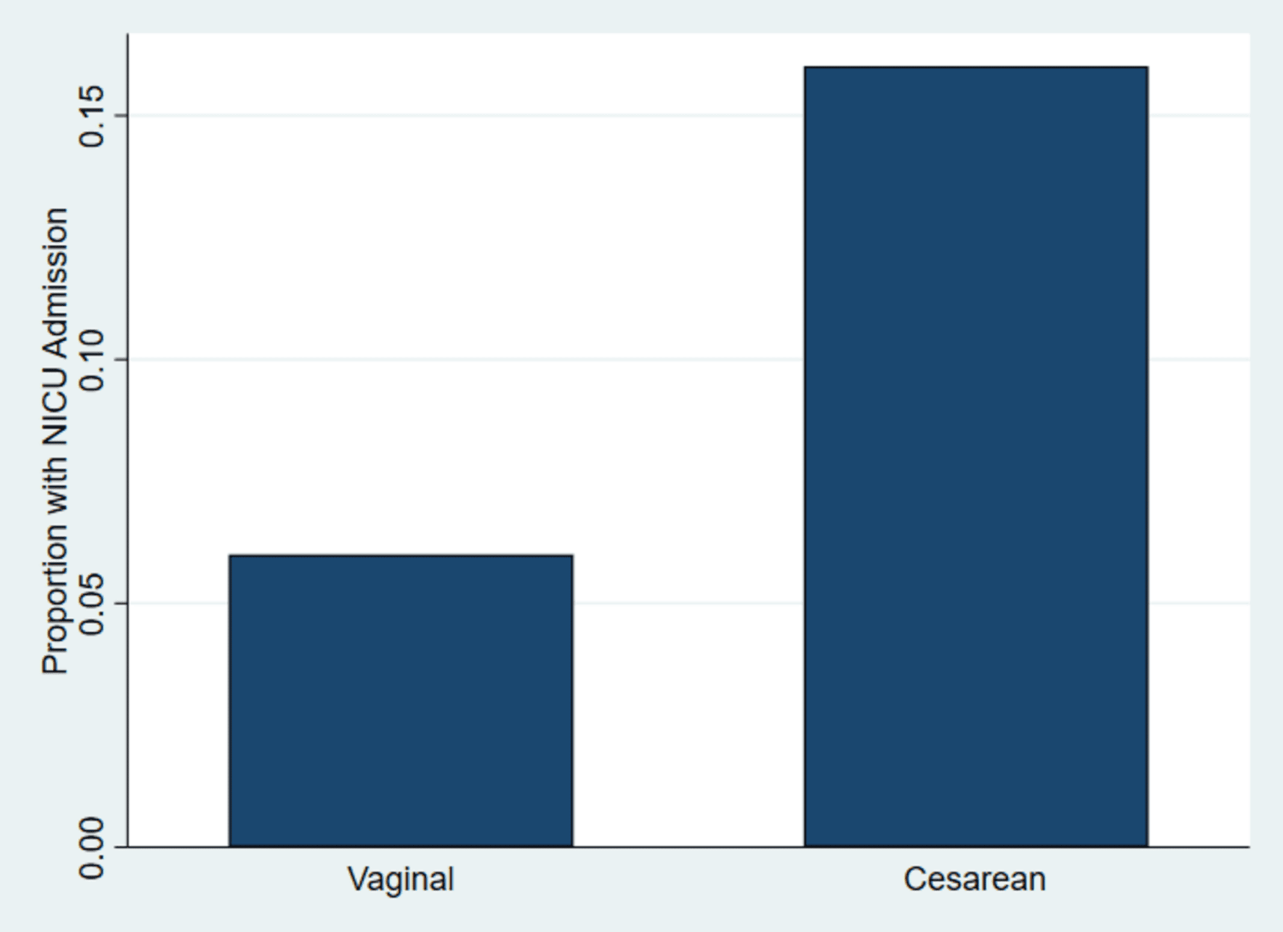
Proportion of Neonatal Intensive Care Unit Admission by Mode of Delivery Source: Figure generated by the authors using Stata version 18 (StataCorp LLC, College Station, TX, USA) [22].

Figure 1 illustrates the proportion of NICU admissions by mode of delivery, complementing the descriptive findings presented in Table 1. The figure provides a visual comparison of NICU admission frequency among infants delivered vaginally and by cesarean section.

From Figure 1, the results indicate a higher proportion of NICU admissions among cesarean deliveries compared with vaginal deliveries. As shown in Table 1, among vaginal deliveries, NICU admission occurred in a smaller proportion of births, while among cesarean deliveries, NICU admission occurred more frequently. The visual pattern in Figure 1 aligns with the descriptive statistics reported in Table 1, which demonstrate higher rates of adverse neonatal indicators among cesarean deliveries, including lower mean gestational age and lower mean birth weight.

Table 2 presents the adjusted associations between mode of delivery and severe maternal morbidity. The table reports adjusted odds ratios (aORs) derived from multivariable logistic regression models that controlled for maternal demographic characteristics, clinical comorbidities, obstetric factors, and calendar year.

**Table 2:** Adjusted Odds Ratios From Multivariable Logistic Regression for Composite Severe Maternal Morbidity (Maternal Blood Transfusion, Uterine Rupture, Unplanned Hysterectomy, or Maternal Intensive Care Unit Admission) by Mode of Delivery Adjusted odds ratios were estimated using multivariable logistic regression, controlling for maternal demographic characteristics, clinical comorbidities, obstetric factors, and calendar year. Robust standard errors were used. + indicates Bachelor’s degree or higher. Source: Table prepared by the authors using Stata version 18 (StataCorp LLC, College Station, TX, USA) [22].

Characteristic	Adjusted odds ratio (95% CI)	p-value
Mode of delivery		
Cesarean delivery vs. vaginal delivery	2.99 (2.94-3.04)	<0.001
Maternal age (per year increase)	1.01 (1.01-1.01)	<0.001
Race		
Black vs. White race	1.16 (1.14-1.18)	<0.001
American Indian or Alaska Native vs. White race	2.09 (1.99-2.19)	<0.001
Asian vs. White race	1.08 (1.05-1.12)	<0.001
Native Hawaiian or Other Pacific Islander vs. White race	1.51 (1.38-1.66)	<0.001
More than one race vs. White race	1.04 (1.00-1.09)	0.054
Ethnicity		
Hispanic vs. Non-Hispanic	0.94 (0.92-0.96)	<0.001
Maternal education		
Less than high school vs. Bachelor's +	1.51 (1.48-1.55)	<0.001
High school vs. Bachelor's +	1.25 (1.23-1.28)	<0.001
Some college/Associate vs. Bachelor's +	1.09 (1.07-1.11)	<0.001
Unknown vs. Bachelor's +	1.77 (1.67-1.88)	<0.001
Maternal clinical factors		
Smoking during pregnancy (Yes vs. No)	1.22 (1.19-1.25)	<0.001
Pre-pregnancy diabetes (Yes vs. No)	1.15 (1.09-1.20)	<0.001
Gestational diabetes (Yes vs. No)	1.00 (0.98-1.03)	0.743
Chronic hypertension (Yes vs. No)	1.70 (1.65-1.75)	<0.001
Gestational hypertension (Yes vs. No)	1.83 (1.80-1.87)	<0.001
Eclampsia (Yes vs. No)	2.97 (2.80-3.14)	<0.001
Prior cesarean delivery (Yes vs. No)	1.01 (0.99-1.02)	0.436
Preterm birth (<37 weeks vs. ≥37 weeks)	2.71 (2.67-2.75)	<0.001
Year of delivery		
2018 vs. 2017	1.03 (1.01-1.05)	0.003
2019 vs. 2017	1.09 (1.07-1.11)	<0.001
2020 vs. 2017	1.11 (1.09-1.13)	<0.001

The findings indicate a strong association between cesarean delivery and severe maternal morbidity. Cesarean delivery was associated with higher adjusted odds of severe maternal morbidity compared with vaginal delivery, with an aOR of 2.99 (95% CI, 2.94-3.04). Increasing maternal age was associated with slightly higher adjusted odds of severe maternal morbidity, with an aOR of 1.01 per year increase (95% CI, 1.01-1.01).

After adjusting for potential confounders, the odds of severe maternal morbidity were highest among American Indian or Alaska Native mothers (OR, 2.09; 95% CI, 1.99-2.19), followed by Native Hawaiian or Other Pacific Islander mothers (OR, 1.51; 95% CI, 1.38-1.66), Black mothers (OR, 1.16; 95% CI, 1.14-1.18), and Asian mothers (OR, 1.08; 95% CI, 1.05-1.12), compared with White mothers.

Hispanic ethnicity was associated with lower adjusted odds of severe maternal morbidity compared with non-Hispanic ethnicity, with an aOR of 0.94 (95% CI, 0.92-0.96). Maternal educational attainment was significantly associated with severe maternal morbidity. Compared with mothers with a bachelor’s degree or higher, the adjusted odds were higher among mothers with less than a high school education (aOR, 1.51; 95% CI, 1.48-1.55), high school graduates (aOR, 1.25; 95% CI, 1.23-1.28), and those with some college education or an associate degree (aOR, 1.09; 95% CI, 1.07-1.11). Mothers with unknown educational attainment also had higher adjusted odds (aOR, 1.77; 95% CI, 1.67-1.88).

Several maternal clinical factors were associated with severe maternal morbidity. Smoking during pregnancy was associated with higher adjusted odds (aOR, 1.22; 95% CI, 1.19-1.25), as was pre-pregnancy diabetes (aOR, 1.15; 95% CI, 1.09-1.20). Gestational diabetes was not significantly associated with severe maternal morbidity after adjustment. Chronic hypertension and gestational hypertension were associated with higher adjusted odds of 1.70 (95% CI, 1.65-1.75) and 1.83 (95% CI, 1.80-1.87), respectively. Eclampsia was associated with markedly higher adjusted odds of severe maternal morbidity (aOR, 2.97; 95% CI, 2.80-3.14). Prior cesarean delivery was not significantly associated with severe maternal morbidity after adjustment.

Preterm birth was associated with substantially higher adjusted odds of severe maternal morbidity (aOR, 2.71; 95% CI, 2.67-2.75). Temporal differences were observed, with higher adjusted odds of severe maternal morbidity in 2018, 2019, and 2020 compared with 2017.

Table 3 presents adjusted associations between mode of delivery and adverse neonatal outcomes. The table reports aORs derived from multivariable logistic regression models that controlled for maternal demographic characteristics, clinical comorbidities, obstetric factors, and calendar year.

**Table 3:** Adjusted Odds Ratios From Multivariable Logistic Regression for Composite Adverse Neonatal Outcomes (Neonatal Intensive Care Unit Admission, Assisted Ventilation at Birth, Assisted Ventilation >6 Hours, or Post-delivery Infant Transfer) by Mode of Delivery Adjusted odds ratios were estimated using multivariable logistic regression, controlling for maternal demographic characteristics, clinical comorbidities, obstetric factors, and calendar year. Robust standard errors were used. Adverse neonatal outcome was defined as a composite of neonatal intensive care unit admission, need for ventilatory support, or infant transfer after delivery. + indicates Bachelor’s degree or higher. Source: Table prepared by the authors using Stata version 18 (StataCorp LLC, College Station, TX, USA) [22].

Characteristic	Adjusted odds ratio (95% CI)	p-value
Mode of delivery		
Cesarean delivery vs. vaginal delivery	2.19 (2.19-2.20)	<0.001
Maternal age (per year increase)	0.995 (0.995-0.996)	<0.001
Race		
Black vs. White race	1.08 (1.08-1.09)	<0.001
American Indian or Alaska Native vs. White race	1.05 (1.03-1.07)	<0.001
Asian vs. White race	0.91 (0.91-0.92)	<0.001
Native Hawaiian or Other Pacific Islander vs. White race	0.99 (0.96-1.02)	0.579
More than one race vs. White race	1.06 (1.05-1.07)	<0.001
Ethnicity		
Hispanic vs. non-Hispanic	0.87 (0.87-0.88)	<0.001
Maternal education		
Less than high school vs. Bachelor's +	1.07 (1.07-1.08)	<0.001
High school vs. Bachelor's +	1.05 (1.04-1.05)	<0.001
Some college/Associate vs. Bachelor's +	1.05 (1.05-1.06)	<0.001
Unknown vs. Bachelor's +	1.21(1.19-1.24)	<0.001
Maternal clinical factors		
Smoking during pregnancy (Yes vs. No)	1.35 (1.34-1.36)	<0.001
Pre-pregnancy diabetes (Yes vs. No)	2.15 (2.11-2.18)	<0.001
Gestational diabetes (Yes vs. No)	1.26 (1.25-1.26)	<0.001
Chronic hypertension (Yes vs. No)	1.55 (1.53-1.56)	<0.001
Gestational hypertension (Yes vs. No)	1.53 (1.52-1.54)	<0.001
Eclampsia (Yes vs. No)	1.50 (1.46-1.54)	<0.001
Preterm birth (<37 weeks vs. ≥37 weeks)	13.87 (13.81-13.93)	<0.001
Year of delivery		
2018 vs. 2017	1.03 (1.03-1.04)	<0.001
2019 vs. 2017	1.06 (1.06-1.07)	<0.001
2020 vs. 2017	1.08(1.07-1.08)	<0.001

The results indicate significant differences in adverse neonatal outcomes by mode of delivery. Cesarean delivery was associated with higher adjusted odds of adverse neonatal outcomes compared with vaginal delivery, with an aOR of 2.19 (95% CI, 2.19-2.20). Increasing maternal age was associated with slightly lower adjusted odds of adverse neonatal outcomes, with an aOR of 0.995 (95% CI, 0.995-0.996).

Adjusted odds of adverse neonatal outcomes varied by maternal race. Compared with White mothers, higher adjusted odds were observed among Black mothers (aOR, 1.08; 95% CI, 1.08-1.09), American Indian or Alaska Native mothers (aOR, 1.05; 95% CI, 1.03-1.07), and mothers reporting more than one race (aOR, 1.06; 95% CI, 1.05-1.07). Lower adjusted odds were observed among Asian mothers (aOR, 0.91; 95% CI, 0.91-0.92). The association for Native Hawaiian or Other Pacific Islander mothers was not statistically significant.

After adjustment for potential confounders, Hispanic mothers had lower adjusted odds of adverse neonatal outcomes compared with non-Hispanic mothers (OR, 0.87; 95% CI, 0.87-0.88). Maternal educational attainment was also associated with adverse neonatal outcomes. Compared with mothers with a bachelor’s degree or higher, the adjusted odds were higher among mothers with less than a high school education (aOR, 1.07; 95% CI, 1.07-1.08), high school graduates (aOR, 1.05; 95% CI, 1.04-1.05), and those with some college education or an associate degree (aOR, 1.05; 95% CI, 1.05-1.06). Mothers with unknown educational attainment also had higher adjusted odds (aOR, 1.21; 95% CI, 1.19-1.24).

Several maternal clinical factors were associated with adverse neonatal outcomes. Smoking during pregnancy was associated with higher adjusted odds (aOR, 1.35; 95% CI, 1.34-1.36). Pre-pregnancy diabetes showed a strong association, with an aOR of 2.15 (95% CI, 2.11-2.18). Gestational diabetes was also associated with higher adjusted odds (aOR, 1.26; 95% CI, 1.25-1.26). Chronic hypertension and gestational hypertension were associated with higher adjusted odds (aOR, 1.55; 95% CI, 1.53-1.56) and (aOR, 1.53; 95% CI, 1.52-1.54), respectively. Eclampsia was associated with higher adjusted odds of adverse neonatal outcomes (aOR, 1.50; 95% CI, 1.46-1.54).

Preterm birth demonstrated the strongest association with adverse neonatal outcomes, with an aOR of 13.87 (95% CI, 13.81-13.93). Temporal differences were observed, with higher adjusted odds of adverse neonatal outcomes in 2018 and 2019 compared with 2017.

## Discussion

In this study, cesarean delivery was associated with substantially higher odds of both severe maternal morbidity and adverse neonatal outcomes compared with vaginal delivery among live births in the United States from 2017 to 2020. After adjustment for maternal age, race, Hispanic origin, clinical conditions, obstetric factors, and calendar year, cesarean delivery showed nearly three-fold higher odds of severe maternal morbidity and more than two-fold higher odds of adverse neonatal outcomes. These findings directly address the study objective and provide population-based evidence on maternal and neonatal outcome patterns associated with mode of delivery.

The observed association between cesarean delivery and severe maternal morbidity aligns with prior literature describing increased risks of surgical and postoperative complications following cesarean birth [5,8]. Severe maternal morbidity components such as transfusion, hysterectomy, uterine rupture, and intensive care admission are more likely to occur in the context of major abdominal surgery and may reflect both procedural risks and underlying obstetric complexity [11,12]. The higher adjusted odds observed in this study persisted after controlling for major maternal comorbidities, suggesting that cesarean delivery itself remains an important marker of elevated maternal risk at the population level.

Maternal demographic factors were also associated with severe maternal morbidity. Higher odds among Black mothers and American Indian or Alaska Native mothers compared with White mothers are consistent with longstanding racial disparities in maternal health outcomes in the United States [1,3,4]. These differences likely reflect a combination of structural, clinical, and health system factors rather than biological differences alone [17]. Hispanic ethnicity was associated with lower adjusted odds of severe maternal morbidity, a pattern that has been described in prior population-based analyses and may relate to differences in baseline risk profiles or unmeasured social factors [19]. Higher maternal educational attainment was associated with lower odds of severe maternal morbidity, supporting the role of socioeconomic factors in shaping maternal health outcomes [6,7].

Several maternal clinical conditions demonstrated strong associations with severe maternal morbidity. Chronic hypertension, gestational hypertension, and eclampsia were each associated with substantially higher odds of severe maternal morbidity in our analysis, with aORs ranging from 1.70 for chronic hypertension to 2.97 for eclampsia. Prior population-based studies have similarly reported approximately two-fold increases in obstetric complications among women with hypertensive disorders of pregnancy (HDPs), supporting the consistency of our findings with established evidence [14,16]. Patients with HDPs have a higher likelihood of cesarean delivery and a significantly increased risk of adverse maternal and neonatal outcomes. HDP is also associated with advancing maternal age, and consistent with our findings, both cesarean delivery rates and adverse outcomes increase with maternal age [14,16]. Pre-pregnancy diabetes was also associated with increased odds, while gestational diabetes was not significantly associated after adjustment, suggesting differential risk profiles between preexisting and pregnancy-related metabolic conditions [15]. Preterm birth showed a strong association with severe maternal morbidity, likely reflecting shared pathways related to obstetric complications and maternal illness severity [18].

For neonatal outcomes, cesarean delivery was associated with higher adjusted odds of adverse neonatal outcomes, defined as a composite of NICU admission, need for ventilatory support, or infant transfer. These findings are consistent with prior reports linking cesarean birth to neonatal respiratory morbidity and early postnatal complications [9,10]. The association persisted after adjustment for gestational age, maternal comorbidities, and demographic factors, suggesting that mode of delivery remains an important correlate of early neonatal health status in population-level data.

Preterm birth demonstrated the strongest association with adverse neonatal outcomes, with markedly elevated adjusted odds. The strong associations with respiratory failure, NICU admission, and early neonatal instability, primarily due to interruption of normal maturation of vital organ systems such as the lungs, central nervous system, and metabolic regulation, with morbidity increasing as gestational age at delivery decreases [15]. To reduce these risks, the American College of Obstetricians and Gynecologists (ACOG) recommends a preventive approach that includes early identification and risk stratification of women at high risk for preterm delivery to allow timely, targeted interventions that may prolong gestation. Recommended strategies include progesterone therapy to maintain uterine quiescence and modulate inflammatory pathways involved in preterm labor, thereby reducing early preterm birth, respiratory morbidity, and NICU utilization. In addition, cervical length screening is recommended for women with a prior preterm birth, with cerclage placement considered for those with a cervical length below 25 mm before 24 weeks’ gestation - an intervention shown to reduce very early preterm births among women at highest risk for respiratory failure and intensive neonatal care [23]. This finding aligns with extensive literature identifying preterm birth as a primary driver of neonatal morbidity and healthcare utilization [15,19]. Maternal conditions such as diabetes, hypertension, smoking during pregnancy, and eclampsia were also associated with higher odds of adverse neonatal outcomes, highlighting the interconnected nature of maternal health and neonatal risk [14,18]. Lower odds observed among Asian mothers and Hispanic mothers are consistent with previously described patterns in national birth data and may reflect differences in baseline risk or obstetric management practices [19,20].

Temporal increases in adjusted odds of both maternal and neonatal adverse outcomes across later study years were observed. These patterns may reflect shifts in obstetric practice, changes in maternal risk profiles, or evolving clinical thresholds for intervention during the study period [2,20]. The use of multiple years of national data strengthens the ability to observe these trends while maintaining population-level generalizability.

Strengths and limitations

This study includes the use of nationally representative CDC Natality Public Use data, a large sample size, standardized variable definitions, and adjustment for a wide range of maternal and obstetric factors. The analysis leveraged robust variance estimation to account for model specification. Limitations include reliance on birth certificate data, which may be subject to misclassification or underreporting for certain clinical conditions [19]. Some variables contained missing data, resulting in variable-specific analytic sample sizes. The cross-sectional design prevents assessment of temporal sequencing and limits interpretation to associations rather than causation. Residual confounding by unmeasured clinical or social factors remains possible. An additional limitation is that the Natality Public Use data do not distinguish between elective and emergency cesarean delivery. Because emergency cesarean deliveries are often performed in the context of acute maternal or fetal complications, the observed associations may partly reflect underlying obstetric risk rather than the delivery mode itself. The inability to stratify by indication or urgency limits more granular assessment of risk by cesarean subtype. In addition, race and Hispanic origin were analyzed as separate variables because the CDC Natality Public Use dataset reports these variables independently rather than as a combined race-ethnicity classification. As a result, our analysis does not distinguish categories such as non-Hispanic White individuals or non-Hispanic Black individuals. This limitation should be considered when interpreting racial and ethnic differences, as the separate treatment of these variables may not fully capture intersectional racial-ethnic patterns in maternal and neonatal outcomes. Future research should explore longitudinal outcomes, incorporate additional clinical detail where available, and examine pathways underlying observed disparities in maternal and neonatal outcomes.

## Conclusions

In this national cross-sectional analysis of more than 15 million births, cesarean delivery was associated with higher adjusted odds of severe maternal morbidity and adverse neonatal outcomes compared with vaginal delivery. These associations persisted after adjustment for maternal demographic characteristics, clinical comorbidities, obstetric factors, and year of delivery. Given the observational design, these findings likely reflect underlying obstetric complexity in addition to delivery mode. The results emphasize the importance of careful risk assessment in delivery planning and highlight the need for continued evaluation of maternal and neonatal outcomes across modes of delivery.
